# Replacing 5-fluorouracil by capecitabine in localised squamous cell carcinoma of the anal canal: systematic review and meta-analysis

**DOI:** 10.3332/ecancer.2016.699

**Published:** 2016-12-01

**Authors:** Karla T Souza, Allan AL Pereira, Raphael L Araujo, Suilane Coelho Ribeiro Oliveira, Paulo M Hoff, Rachel P Riechelmann

**Affiliations:** 1 Department of Radiology and Oncology, Instituto do Cancer do Estado de São Paulo, São Paulo 01246-000, Brazil; 2 Department of Upper Gastrointestinal and Hepato-Pancreato-Biliary Surgery, Barretos Cancer Hospital, Barretos 14784-400, Brazil; 3 Universidade Estadualdo Piaui, Teresina 64049522, Brazil

**Keywords:** capecitabine, anal canal neoplasm, anus neoplasm, anal cancer, chemoradiation

## Abstract

**Background:**

The standard treatment for localised squamous cell carcinoma of the anal canal (SCCAC) is chemoradiotherapy (CRT) with infusional 5-fluorouracil (5-FU) and mitomycin. Because 5-FU and capecitabine have offered similar efficacy in many phase-III trials of solid tumours, studies have tested capecitabine in this setting of SCCAC. However, these studies are small and have reported variable results. Therefore, a systematic review and meta-analysis was performed.

**Methods:**

Medline, Scopus and Embase were searched for studies that evaluated the efficacy outcomes of capecitabine used as a substitute of 5-FU in the CRT of localised SCCAC. The primary endpoint was complete response rate (CRR) at 6 months. Metaprop analysis of reported CRR-based on pooled estimates of proportions with corresponding 95% confidence intervals (95%CI) were calculated on the base of the Freeman-Tukey double arcsine transformation.

**Results:**

We retrieved 300 studies, of which six met our eligibility criteria. The capecitabine dose ranged from 500 mg/m^2^ to 825 mg/m^2^ BID for 5 days per week during radiation. With a total of 218 patients, the median follow-up was 21.5 months (14–23). The pooled analysis of three trials (*N* = 132 patients) reported a CRR at 6 months of 88% (83%–94%), considering all clinical stages. The pooled analysis of overall CRR (*N* = 218 patients), evaluated at different intervals, showed an overall CRR of 91% (87%–95%). Rates of locoregional relapse varied from 3.2% to 21%. The majority of patients completed the planned radiotherapy dose (93.5%–100%) and any chemotherapy interruption was reported in up to 55.8% of patients.

**Conclusions:**

Capecitabine is an acceptable and more convenient alternative to infusional 5-FU in the CRT for localised SCCAC, offering similar clinical CRR to those reported by phase-III trials.

## Introduction

Squamous cell carcinoma of the anal canal (SCCAA) is an uncommon cancer in developed countries, accounting for 1–5% of digestive system malignancies [1]. The risk factors associated with this disease are human papillomavirus (HPV) infection, lifetime number of sexual partners, cigarette smoking, and infection with human immunodeficiency virus (HIV) [2–4].

The standard treatment for localised SCCAC is definitive chemoradiotherapy with infusional 5-fluorouracil (5-FU) and mitomycin, proposed by Nigro in 1974 [5]. Two European phase-III trials evaluated the role of chemotherapy added to radiotherapy in comparison to radiotherapy alone, and they have proven the superiority of the combined treatment for locoregional control [6, 7]. The need for mitomycin was also addressed by the landmark RTOG 98-11 phase-III trial, which showed that adding this drug led to better colostomy-free survival and disease-free survival [8]. After long-term follow-up, this trial also demonstrated that the addition of mitomycin to chemoradiation was associated with improved 5-year overall survival (78.3 vs. 70.7%, *p* = 0.026) over neoadjuvant cisplatin and 5-FU followed by chemoradiation with cisplatin [9]. The ACT-II phase III, in contrast, demonstrated similar efficacy results for chemoradiation with mitomycin or cisplatin [10]. Recently, intensity modulated radiation therapy (IMRT) has been tested to treat localised SCCAC, offering improvement in terms of reduced toxicity [11].

Capecitabine is an oral fluoropyrimidine that has been evaluated in many solid tumours as a more convenient substitute to infusional 5-FU, with proven non-inferior efficacy in the adjuvant and metastatic settings of colorectal cancer [12–14] and gastric cancer [15, 16]. Regarding its role in radiosensibilisation, capecitabine provides similar results in localised rectal adenocarcinoma, with non-inferior 5-year overall survival (76 vs. 67%, *p* = 0.004), similar local recurrence (6 vs. 7%, *p* = 0.67) and similar all grade diarrhoea (24 vs. 22%, *p* = 0.67), when compared with infusional 5-FU [17]. One of the aims of non-inferiority trials is to test drugs with similar effectiveness but with more convenient treatment schedules in comparison with the standard of care [18]. Capecitabine fulfils this requirement, particularly in anal cancer, where patients often need to have a central catheter insertion or hospitalisations when infusion pumps are not available, a common situation in middle- and low-income countries.

Capecitabine has been assessed as a replacement for 5-FU in retrospective studies and phase-II trials in the treatment of SCCAC, including a phase II conducted by our group [19]. However, there is insufficient evidence to recommend this substitution to date. Therefore, we performed a systematic review and meta-analysis of published studies to better estimate the magnitude of benefit and treatment outcomes of capecitabine in the setting of localised SCCAC.

## Materials and methods

### Search and selection criteria

We performed a systematic review of studies that evaluated the efficacy outcomes of capecitabine used as a substitute of 5-FU in the chemoradiotherapy of localised SCCAC. There were no restrictions on the inclusion of retrospective series, observational cohorts, phase-I, phase–II, or phase-III trials, and there were not placed restrictions on the chemotherapy drug combined with capecitabine or the type of radiotherapy used (3-dimensional conformal technique or IMRT). The review adheres to the guidelines outlined by the PRISMA statement [20].

Articles were screened for eligibility by two independent investigators (KTS and AALP) followed by a third blinded reviewer (RPR) in case of divergence. The search for full publications was conducted through PubMed, EMBASE, and Scopus. There were no language restrictions. We also hand-searched the reference lists of included studies for additional publications.

We sought eligible trials in the EMBASE and Scopus Database including the following terms: ‘anal cancer’ OR ‘anal neoplasm’ OR ‘anal carcinoma’ OR ‘anus cancer’ OR ‘anus neoplasm’ OR ‘anus carcinoma’ AND ‘capecitabine’, from the date on inception onward until August 2015. Pubmed database was conducted using the terms already described and the MeSH descriptor ‘anus neoplasm’, from the date on inception onward until December 2015.

### Data extraction

We extracted the following data independently, using standardised collection forms: study design, number of patients included, primary and secondary outcomes, chemotherapy and radiotherapy regimens, doses and respective doses reduction or interruption, median follow-up, age, stage, performance status, grade 3 or 4 toxicities, and complete response rates and locoregional relapse rates. Some authors of the eligible studies were contacted in order to provide relevant unpublished data.

### Outcome measures

Our primary endpoint was complete response rate (CRR) at 6 months. We chose CRR at 6 months as our main endpoint because this has been shown to be a better predictor of response than early evaluations [21] and because it was an outcome reported by most eligible studies. Secondary endpoints were rates of locoregional relapse, interruptions in chemotherapy and radiotherapy and toxicity. Data about adverse events were extracted as they were reported, without a standardised manner, given the variability of this information across trials.

### Analysis and synthesis

Descriptive statistics were used to summarise the study characteristics. Metaprop analysis of reported CRR based on pooled estimates of proportions with corresponding 95% confidence intervals (95%CI) were calculated on the base of the Freeman-Tukey double arcsine transformation [22, 23]. Respective 95% confidence intervals (CI) were calculated for each estimate and presented in forest plots. The pooled odds ratio (OR), symbolised by a solid diamond at the bottom of the forest plot (the width of which represents the 95% CI) is the best estimate of the pooled outcome. All analyses were performed by STATA 13 statistical software (StataCorp, College Station, TX, USA).

## Results

### Results of search strategy

**Figure 1.**
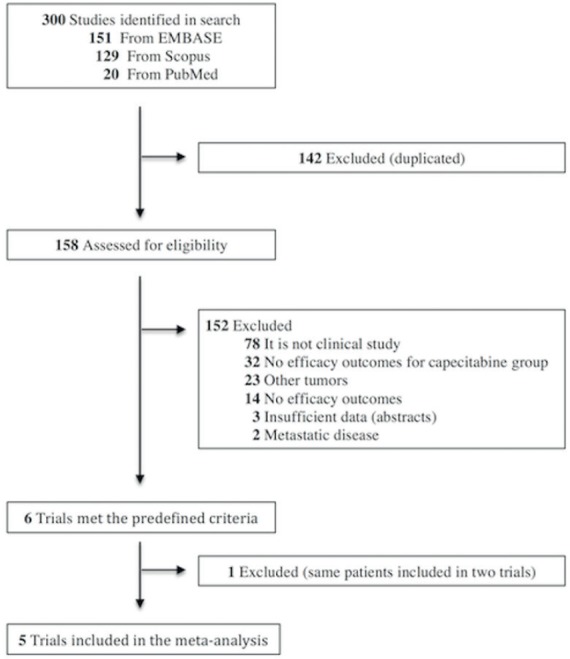
Trial selection process.

The search resulted in 300 entries, of which 142 duplicated records were removed. From the remaining 158 studies, 152 were excluded as shown in Figure 1. Six trials met the predefined criteria [19, 24–28]; however, patients from the phase-I trial by Deenen *et al* [24] were also included in the analysis of Meulendijks’s retrospective trial [28], and for this reason, the former was excluded. Five trials were included in the pooled analysis, totalling 218 patients [19, 25–28].

### Characteristics of eligible trials

The characteristics of the five eligible studies are summarised in Table 1. There were two phase-II trials [19, 25], both open-label prospective single-arm studies, and three retrospective studies, with one of them being a comparative study [28]. The number of patients per study ranged from 24 to 62, and the median follow-up periods ranged from 14 to 23 months. The percentage of T3 or T4 stage was similar across studies, while the rate of positive clinical lymph nodes varied from 25.8% to 65.5%. All trials included complete response rate as one of its primary endpoints, but time intervals for response evaluation differed across studies. The capecitabine dose ranged from 500 mg/m^2^ to 825 mg/m^2^ BID for 5 days per week during radiation. All studies tested capecitabine as replacement for 5-FU in the Nigro regimen, except one where capecitabine was combined with cisplatin [26]. Patients were treated with a 3-dimensional conformal technique or IMRT technique, with radiation doses ranging from 50.4 Gy to 59.4 Gy.

### Complete response rates

**Figure 2.**
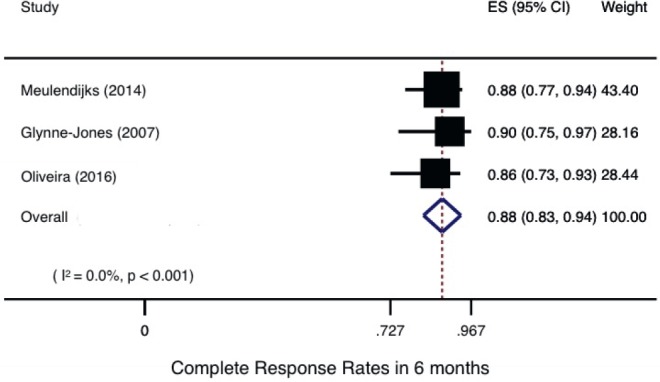
Complete response rates in 6 months.

All trials reported CRR, although response evaluations differed across them: one trial [28] evaluated response only based on anal clinical examination, while the others three studies [19, 25, 26] included abdominal and pelvis imaging tests and one trial did not describe methods of response assessment [27]. Only three trials described CRR at 6 months [19, 25, 28]. The pooled analysis of these trials, with a total of 132 patients, reported a CRR at 6 months of 88% [83%–94%], considering all clinical stages (Figure 2).

**Figure 3.**
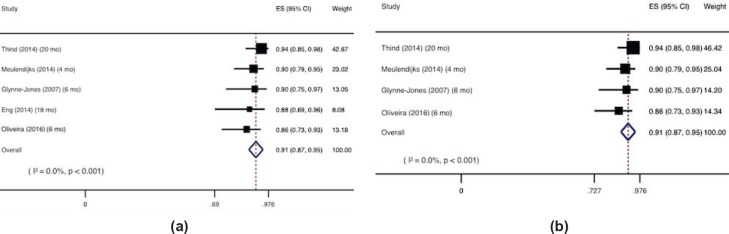
Overall complete response: (a) All studies, (b) Studies with mitomycin and capecitabine only.

The pooled analysis of all overall CRR as reported in each study, evaluated at different time points (ranging from 1 to 6 months) and including all the 218 patients, showed an overall CRR of 91% (87%–95%) (Figure 3a). This finding remained the same after sensitive analysis, excluding the study with cisplatin, with a total of 194 patients [19, 25, 27, 28] (Figure 3b).

### Secondary endpoints

After a median follow-up that ranged from 14 to 23 months, locoregional relapse, defined as primary or nodal recurrence, was reported in 3.2% to 21%. Disease progression with distance metastases was uncommon, ranging from 0% to 3%. Clinical follow-up was initially every 3 months for one to two years, according to each trial, and then, every 6 months up to 5 years. In the fourth and fifth years of follow-up, the frequency of clinical evaluations varied across studies, from semiannual [25, 26] to annual [19, 28] evaluations. Only the Eng *et al*. trial recommended follow-up with images that were done annually during all follow-up [26]. One of the studies did not describe their recommendations for follow-up [27].

With respect to treatment compliance, chemotherapy interruptions were reported in up to 55.8% of patients and dose reductions were necessary in up to 20.9%.

The majority of patients completed the planned radiotherapy dose [93.5%–100%]; however, temporary pauses were required in up to 25.6% of patients. The most common reason for these interruptions was treatment toxicity, mainly dermatitis, and the longest pause was 14 days.

A total of 194 patients were available for acute treatment toxicity [19, 25, 27, 28]. The remaining 24 patients were not included because in Eng's study the toxicities were reported for all the patients included in the trial, without specifying the toxicities of the subgroup that received capecitabine [26]. Perianal dermatitis was the most common toxicity, experienced in varying degrees, and 23.2% to 63.3% of patients had grade 3 or 4 dermatitis. Other commons grade 3 and 4 toxicities were hematologic (5.1% to 16.3%) and diarrhoea (3.2% to 7.5%) (Table 1).

The present systematic review and meta-analysis evaluated the substitution of infusional 5-FU for capecitabine in the treatment of localised anal canal cancer. Although there is not any randomised trial, the pooled analysis of uncontrolled studies showed a CRR at 6 months of 88% and an overall CRR of 91% for patients who used capecitabine. This result is similar to the 90.5% of CRR at week 26 with mitomycin and infusional 5-FU reported by ACT-II phase-III trial [10].

After the publication by Nigro *et al*. [5] in the mid-1970s, chemoradiotherapy became the standard treatment for SCCAC. The combination of radiotherapy with infusional 5FU and mitomycin is considered the optimal regimen for these patients, with complete response rate (by biopsy) of 92% after 4–6 weeks after treatment [29]. In the ACT-II phase-III trial, which tested cisplatin against mitomycin, waiting until 6.5 months after treatment completion to evaluate response led to an increase in CRR, from 65.6 at week 11 to 83.5% at week 26 [10, 21]. Since then, the National Comprehensive Network on Cancer guideline recommends that patients with localised anal cancer who were treated with chemoradiation be considered in complete remission not until 6 months after treatment [30]. Based on these premises, we defined our primary endpoint as CRR at 6 months from treatment completion.

Four studies included in this meta-analysis used capecitabine in combination with mitomycin [19, 25, 27, 28], while one trial evaluated the same drug in combination with cisplatin [26]. Although patients in the Eng *et al*. study received cisplatin in combination with 5-FU or capecitabine, this trial was included in the meta-analysis, because in the ACT-II trial cisplatin and mitomycin provided similar CRR [10].

The median age ranged from 57 to 61 years and in all trials there was a predominance of women, which is according to the epidemiological profile of this disease. Three studies included HIV patients [19, 26, 27], and the phase-II trial conducted by our group was the one with the highest prevalence of HIV-positive patients (9.3%). In that trial, HIV positivity was not associated with inferior CRR at 6 months, 87% for HIV-negative patients and 75% for HIV-positive patients, without statistical difference (unpublished data). At least half of patients from the studies included presented more advanced disease, T3/4 or N+; in the RTOG 98-11 trial, 35% of patients had T3/4 tumours and 26% were N+ (8).

The rate of locoregional recurrence during the studies periods varied across studies, from 3.2% to 21%. The outlier locoregional recurrence rate, when compared to the others, was present in the retrospective study conducted by Meulendijks *et al*., which showed locoregional relapse in 12 of 58 patients. In the remaining studies, this rate did not exceed 10% of patients. One explanation for these findings is the higher prevalence of patients with more advanced stage in the Meulendijks *et al*.: 72% had stage III, while in other studies this index did not exceed 60% in other studies.

Although there were a large number of patients who temporarily discontinued capecitabine because of toxicity, a high incidence of interruptions (55.8%) was seen in only one trial, while the rate of interruptions was lower than 20% in the others. One of the reasons that may have influenced these findings in Oliveira's study was the largest prevalence of HIV-positive patients. It may have led to higher incidence of toxicities (this was the trial with the higher incidence of haematologic toxicity) and consequently, more chemotherapy interruptions; and another possible reason was the higher dose of mitomycin (15 mg/m^2^) utilised compared to other studies. The adherence to radiotherapy was relatively homogeneous, with more than 93% of the patients completing the planned dose schedules, which is in agreement with the 91% planned dose completion reported previously in studies with infusional 5-FU [6].

Among grade 3 or 4 adverse events, dermatitis was the most common, as expected (Table 1), and its incidence is similar to that reported in the literature for infusional 5-FU in this context [7, 9]. Haematological toxicity and diarrhoea have also been reported, however, with lower incidence than previously described for infusional 5-FU – 61.8% for grade 3 and 4 haematologic toxicity and 36.6% for grade 3 and 4 diarrhoea [9]. These lower rates likely reflect more flexibility in dose reduction and/or interruptions performed in real life (retrospective studies). There were no quality of life analyses in any of the studies included.

Given that this was a meta-analysis of uncontrolled published studies, some limitations must be addressed. First, we were unable to retrieve the individual patient data, which would have allowed us to pool the data from all studies, to look into the efficacy of capecitabine across different clinical stages, gender, and HIV status, to combine the CRR at other time points, and to better evaluate treatment-related toxicities. Second, there were differences in study designs, which is a source of heterogeneity in our analysis (retrospective vs. prospective studies). Third, the evaluation of response, including CRR, varied among studies. Finally, the lack of randomised data decreases the quality of evidence yield from the present meta-analysis. However, this is still the best available evidence on the use of capecitabine in definitive chemoradiation for patients with localised anal cancer. Given the rarity of anal cancer and the results of phase-III trials that have demonstrated the noninferiority of capecitabine to 5FU in other solid tumours, it is very unlikely that a phase-III trial be developed in this scenario. Indeed, we do not think that would be necessary.

## Conclusions

Despite being a rare cancer, the incidence of anal cancer has increased and the search for therapeutic alternatives that maintain the effectiveness but are more convenient should be encouraged. The replacement of infusional 5-FU with capecitabine is one such example and in clinical practice it is already widely adopted. Based on this meta-analysis, we think that capecitabine is an acceptable and more convenient alternative to infusional 5-FU in the chemoradiotherapy for localised squamous cell carcinoma of the anal canal.

## Figures and Tables

**Table 1.** Selected trials characteristics.

Characteristics	Glynne-Jones, 2007	Eng, 2013	Thind, 2014	Meulendijks, 2014	Oliveira, 2016
**Number of pts**	31	24^a^	62^b^	58	43
**Age-median**	61 years	NR	60 years	59 years	57 years
**Gender % (male:female)**	45:55	NR	38:62	38:62	28:72
**Type of trial**	Prospective single arm phase II	retrospective	retrospective	retrospective, comparative	prospective single arm phase II
**Chemotherapy regimen**	mmc 12 mg/m^2^ d1 + cap 825mg/m^2^bid	cis 20 m/m^2^ d1 orcis 4 mg/m^2^/d + cap 825 mg/m^2^bid	mmc 12 mg/m^2^ d1 + cap 825 mg/m^2^bid	mmc 10 mg/m^2^ d1 + cap 500 -825 mg/m^2^ bid	mmc 15 mg/m^2^ d1 + cap 825 mg/m^2^bid
**Chemotherapy interruptions**	6 (19.3%)	NR	0	8 (13.8%)	24 (55.8%)
**Completed radiotherapy dose**	29 (93.5%)	NR	65 (98.5%)^c^	58 (100%)	43 (100%)
**Stage I**	NR	NR	26 (39%)^c^	0	0
**Stage II**	NR	NR	15 (23%)^c^	14 (24%)^d^	17 (40%)
**Stage III**	NR	NR	25 (38%)^c^	42 (72.4%)^d^	26 (60%)
**T stage**					
**T1**	6 (19.3%)	NR	NR	0	0
**T2**	9 (29%)	NR	NR	29 (50%)^d^	16 (37.2%)
**T3**	12 (38.7%)	NR	NR	19 (32.7%)^d^	16 (37.2%)
**T4**	4 (13%)	NR	NR	10 (17.2%)^d^	11 (25.5%)
**N stage negative**	23 (74.2%)	NR	NR	18 (31%)^d^	22 (51%)
**N stage positive**	8 (25.8%)	NR	NR	38 (65.5%)^d^	21 (49%)
**HIV status**	NR	NR	1 (2%)	4 (7%)	4 (9%)
**Overall CRR**	90% in 6 months	83.3% in 4.5 months	93.5% in 20 months	87% in 6 months	86% in 6 months
**OS**	93.5% in 14 months	NR	95% in 20 months	86.2% in 36 months	97.7% in 23 months
**Locoregional relapse**	3 (9.7%)	NR	2 (3.2%)	21%	3 (7%)
**Dermatitis G3/4**	12 (38.7%)	NR	42 (63.6%)^c^	18 (31%)	10 (23%)
**Diarrhoea G3/4**	1 (3.2%)	NR	5 (7.5%)^c^	2 (3.4%)	2 (4.6%)
**Haematologic G3/4**	2 (4.6%)	NR	NR	3 (5%)	7 (16%)

a Subgroup that used capecitabine.

b excluded 4 patients that had adenocarcinoma or adenoma.

c included all patients of the study (SCCAC, adenocarcinoma and adenoma).

d 2 patients without definition of stage.

cap, capecitabine; cis, cisplatin; CRR, complete response rate; HIV, human immunodeficiency virus; mmc, mitomycin; NR, not reported; OS, overall survival.

## References

[1] Johnson LG, Madeleine MM, Newcomer LM (2004). Anal cancer incidence and survival: the surveillance, epidemiology, and end results experience, 1973-2000. Cancer.

[2] Siegel RL, Miller KD, Jemal A (2016). Cancer statistics, 2016. CA Cancer J Clin.

[3] Palefsky JM (1994). Anal human papillomavirus infection and anal cancer in HIV-positive individuals: an emerging problem. Aids.

[4] Shiels MS, Pfeiffer RM, Chaturvedi AK (2012). Impact of the HIV epidemic on the incidence rates of anal cancer in the United States. J Natl Cancer Inst.

[5] Nigro ND, Vaitkevicius VK, Considine B (1974). Combined therapy for cancer of the anal canal: a preliminary report. Dis Colon Rectum.

[6] UK Co-ordinating Committee on Cancer Research (1996). Epidermoid anal cancer: results from the UKCCCR randomised trial of radiotherapy alone versus radiotherapy, 5-fluorouracil, and mitomycin UKCCCR Anal Cancer Trial Working Party. Lancet.

[7] Bartelink H, Roelofsen F, Eschwege F (1997). Concomitant radiotherapy and chemotherapy is superior to radiotherapy alone in the treatment of locally advanced anal cancer: results of a phase III randomized trial of the European Organization for Research and Treatment of Cancer Radiotherapy and Gastrointestinal Cooperative Groups. J Clin Oncol.

[8] Ajani JA, Winter KA, Gunderson LL (2008). Fluorouracil, mitomycin, and radiotherapy vs fluorouracil, cisplatin, and radiotherapy for carcinoma of the anal canal: a randomized controlled trial. JAMA.

[9] Gunderson LL, Winter KA, Ajani JA (2012). Long-term update of US GI intergroup RTOG 98-11 phase III trial for anal carcinoma: survival, relapse, and colostomy failure with concurrent chemoradiation involving fluorouracil/mitomycin versus fluorouracil/cisplatin. J Clin Oncol.

[10] James RD, Glynne-Jones R, Meadows HM (2013). Mitomycin or cisplatin chemoradiation with or without maintenance chemotherapy for treatment of squamous-cell carcinoma of the anus (ACT II): a randomised, phase 3, open-label, 2 x 2 factorial trial. The Lancet. Oncol.

[11] Kachnic LA, Winter K, Myerson RJ (2013). RTOG 0529: a phase 2 evaluation of dose-painted intensity modulated radiation therapy in combination with 5-fluorouracil and mitomycin-C for the reduction of acute morbidity in carcinoma of the anal canal. Int J Radiat Oncol, Biol, Phys.

[12] Twelves C, Wong A, Nowacki MP (2005). Capecitabine as adjuvant treatment for stage III colon cancer. N Engl J Med.

[13] Hoff PM, Ansari R, Batist G (2001). Comparison of oral capecitabine versus intravenous fluorouracil plus leucovorin as first-line treatment in 605 patients with metastatic colorectal cancer: results of a randomized phase III study. J Clin Oncol.

[14] Rothenberg ML, Cox JV, Butts C (2008). Capecitabine plus oxaliplatin (XELOX) versus 5-fluorouracil/folinic acid plus oxaliplatin (FOLFOX-4) as second-line therapy in metastatic colorectal cancer: a randomized phase III noninferiority study. Ann Oncol.

[15] Kang YK, Kang WK, Shin DB (2009). Capecitabine/cisplatin versus 5-fluorouracil/cisplatin as first-line therapy in patients with advanced gastric cancer: a randomised phase III noninferiority trial. Ann Oncol.

[16] Cunningham D, Starling N, Rao S (2008). Capecitabine and oxaliplatin for advanced esophagogastric cancer. N Engl J Med.

[17] Hofheinz RD, Wenz F, Post S (2012). Chemoradiotherapy with capecitabine versus fluorouracil for locally advanced rectal cancer: a randomised, multicentre, non-inferiority, phase 3 trial. Lancet. Oncol.

[18] Riechelmann RP, Alex A, Cruz L (2013). Non-inferiority cancer clinical trials: scope and purposes underlying their design. Ann Oncol.

[19] Oliveira SC, Moniz CM, Riechelmann R (2016). Phase II study of capecitabine in substitution of 5-FU in the chemoradiotherapy regimen for patients with localized squamous cell carcinoma of the anal canal. J Gastrointest Cancer.

[20] Moher D, Liberati A, Tetzlaff J (2009). Preferred reporting items for systematic reviews and meta-analyses: the PRISMA statement. J Clin Epidemiol.

[21] Glynne-Jones R, James R, Meadows H (2012). Optimum time to assess complete clinical response (CR) following chemoradiation (CRT) using mitomycin (MMC) or cisplatin (CisP), with or without maintenance CisP/5FU in squamous cell carcinoma of the anus: results of ACT II. ASCO Meeting Abstracts.

[22] Freeman MF, Tukey JW (1950). Transformations related to the angular and the square root.

[23] Nyaga VN, Arbyn M, Aerts M (2014). Metaprop: a Stata command to perform meta-analysis of binomial data. Arch Public Health.

[24] Deenen MJ, Dewit L, Boot H (2013). Simultaneous integrated boost-intensity modulated radiation therapy with concomitant capecitabine and mitomycin C for locally advanced anal carcinoma: a phase 1 study. Int J Radiat Oncol Biol Phys.

[25] Glynne-Jones R, Meadows H, Wan S (2008). EXTRA–a multicenter phase II study of chemoradiation using a 5 day per week oral regimen of capecitabine and intravenous mitomycin C in anal cancer. Int J Radiat Oncol Biol Phys.

[26] Eng C, Chang GJ, You YN (2013). Long-term results of weekly/daily cisplatin-based chemoradiation for locally advanced squamous cell carcinoma of the anal canal. Cancer.

[27] Thind G, Johal B, Follwell M (2014). Chemoradiation with capecitabine and mitomycin-C for stage I-III anal squamous cell carcinoma. Radiat Oncol.

[28] Meulendijks D, Dewit L, Tomasoa NB (2014). Chemoradiotherapy with capecitabine for locally advanced anal carcinoma: an alternative treatment option. Br J Cancer.

[29] Flam M, John M, Pajak TF (1996). Role of mitomycin in combination with fluorouracil and radiotherapy, and of salvage chemoradiation in the definitive nonsurgical treatment of epidermoid carcinoma of the anal canal: results of a phase III randomized intergroup study. J Clin Oncol.

[30] National Comprehensive Cancer Network (NCCN) (2016). http://www.nccn.org/professionals/physician_gls/pdf/anal.pdf.

